# Putting the brakes on muscle growth: Myostatin as regulator of disuse atrophy

**DOI:** 10.1113/EP093732

**Published:** 2026-07-26

**Authors:** Derk H. C. Nieuwenhuijsen, Moritz Eggelbusch, Rob C. I. Wüst

**Affiliations:** ^1^ Department of Human Movement Sciences, Faculty of Behavioural and Movement Sciences Vrije Universiteit Amsterdam, Amsterdam Movement Sciences Amsterdam the Netherlands; ^2^ Professorship of Exercise Biology, TUM School of Medicine and Health Technical University of Munich Munich Germany

**Keywords:** myostatin, physical inactivity, protein degradation, protein synthesis, satellite cells, skeletal muscle

## Abstract

Skeletal muscle is a highly plastic tissue that rapidly adapts to changes in mechanical loading and metabolic activity. Periods of inactivity, including bed rest, limb immobilization or microgravity, induce a pronounced loss of muscle mass and function. This review examines the mechanistic role of myostatin (growth differentiation factor‐8; GDF‐8), a member of the transforming growth factor‐β superfamily, in mediating inactivity‐induced skeletal muscle atrophy. Accumulating evidence from human and rodent studies demonstrates that physical inactivity upregulates myostatin expression and signalling, shifting muscle protein turnover toward net protein degradation. Mechanistically, myostatin binds to the activin type IIB receptor (ActRIIB) and activates Smad2/3 signalling, which suppresses Akt phosphorylation and downstream mTOR activity, resulting in reduced protein translation. Diminished Akt signalling activates FoxO transcription factors, promoting ubiquitin–proteasome‐mediated proteolysis. In parallel, myostatin maintains satellite cells in a quiescent state, impairing MyoD‐driven activation and limiting myogenesis, thereby reducing regenerative capacity during and after physical inactivity. We provide a narrative mini‐review on the time course of gene expression of myostatin during inactivity. Finally, these mechanistic insights have stimulated therapeutic strategies targeting the myostatin–ActRIIB axis, notably bimagrumab, a monoclonal antibody against ActRIIB and inhibitor of downstream myostatin signalling. Evidence from human and rodent studies suggests that myostatin inhibition may represent a promising strategy to counteract skeletal muscle disuse atrophy caused by inactivity. Collectively, the current evidence highlights myostatin as a central molecular integrator of mechanical unloading‐induced muscle atrophy.

## Abstract (highlights)

- **What is the topic of this review?**
  This review discusses how physical inactivity (e.g., bed rest, immobilization, microgravity) upregulates myostatin and how myostatin contributes to skeletal muscle disuse atrophy, integrating evidence from molecular, cellular and human in vivo experiments.
- **What advances does it highlight?**
  The review highlights myostatin as a central coordinator of inactivity‐induced muscle loss through Smad2/3‐mediated suppression of Akt–mTOR signalling, activation of proteolysis and inhibition of satellite cell activation, and discusses translational implications and safety considerations of myostatin‐targeted therapies in the clinic but also spaceflight.

## 1 INTRODUCTION

Skeletal muscle comprises ∼45% of human body mass and is essential for movement and force production. It is a highly metabolic and plastic tissue that adapts its size and metabolism to environmental and functional demands. Skeletal muscle plays a central role in whole‐body metabolic homeostasis, serving as the primary site of nutrient utilization and as an endocrine organ that releases myokines with local and systemic effects (Boardman et al., 2023; Flück & Hoppeler, 2003; Pedersen, 2011; Sartori et al., 2021).

Physical inactivity is highly prevalent and represents a major public health concern (Guthold et al., 2018). Reduced mechanical and metabolic loading during periods of physical inactivity leads to skeletal muscle atrophy and metabolic deterioration (Eggelbusch et al., 2024; Hendrickse et al., 2022), and low muscle mass is independently associated with multiple chronic diseases and increased mortality risk (Srikanthan & Karlamangla, 2014). Physical inactivity is unavoidable in numerous clinical and extreme settings, such as bed rest, limb immobilization, prolonged sedentary behaviour and exposure to microgravity (Bosutti et al., 2025; Dirks et al., 2018; Ferrando et al., 1996). Muscle deconditioning is characterized by a reduction in muscle cross‐sectional area, decreased force‐generating capacity, impaired metabolic flexibility and reduced insulin sensitivity (Bosutti et al., 2025; Kortebein et al., 2007), which can already occur after short periods of inactivity (Eggelbusch et al., 2024; Hendrickse et al., 2022). At the molecular level, disuse atrophy results from a sustained imbalance between muscle protein synthesis and degradation, leading to net protein loss (Rudrappa et al., 2016). Clinically, skeletal muscle loss is often associated with impaired physical recovery, increased risk of complications during hospitalization, reduced functional independence and elevated mortality risk (Jang et al., 2025).

A central molecular regulator of skeletal muscle mass is myostatin (growth differentiation factor‐8; GDF‐8), a member of the transforming growth factor‐β (TGF‐β) superfamily (Hoogaars & Jaspers, 2018; McPherron et al., 1997). Myostatin acts as a potent negative regulator of muscle growth, and its role in constraining skeletal muscle mass is well established (McPherron et al., 1997). Accumulating evidence from both human and animal studies indicates that myostatin expression and signalling are upregulated during periods of mechanical unloading and physical inactivity (Carlson et al., 1999; Gustafsson et al., 2010; Zachwieja et al., 1999). This suggests that myostatin contributes to inactivity‐induced skeletal muscle atrophy (Hanson et al., 2023; Hoogaars & Jaspers, 2018; Smith et al., 2020) and associated impairments in muscle regeneration and metabolism (Ahmad et al., 2023; McCroskery et al., 2003). Here, we describe the role of myostatin in relation to the molecular mechanisms underlying skeletal muscle atrophy during physical inactivity. Specifically, this narrative mini‐review aims to summarize current evidence demonstrating how increased, disuse‐induced myostatin signalling contributes to skeletal muscle loss through several interconnected pathways, including (1) suppression of protein synthesis and potential induction of anabolic resistance, (2) stimulation of proteolysis, and (3) inhibition of satellite cell activation and proliferation. We first discuss skeletal muscle adaptations to physical inactivity and the canonical myostatin signalling pathway, followed by evidence for inactivity‐induced upregulation of myostatin and its consequences for muscle protein turnover and regenerative capacity. Finally, we discuss the potential clinical implications and future perspectives of targeting myostatin signalling during disuse.

## 2 SKELETAL MUSCLE ALTERATIONS DURING PHYSICAL INACTIVITY

Periods of physical inactivity, such as microgravity exposure or prolonged bed rest, are characterized by a marked reduction in mechanical loading (Bosutti et al., 2025). The physiological and molecular consequences of skeletal muscle inactivity develop progressively over time (Ferrando et al., 1996; Pang et al., 2023; Smith et al., 2020). The earliest and dominant response is a suppression of muscle protein synthesis, accompanied by the development of anabolic resistance, whereby skeletal muscle displays a blunted anabolic response to amino acids and insulin despite adequate nutrient availability (Kilroe et al., 2020; Phillips & McGlory, 2014; Rudrappa et al., 2016). This impaired anabolic sensitivity is associated with reduced Akt–mechanistic target of rapamycin complex 1 (mTORC1) signalling and diminished translational efficiency (Francaux et al., 2016), and can develop within days of unloading (Kilroe et al., 2020).

During short‐term inactivity (<10 days), reductions in myofibrillar protein synthesis rates account for most of the negative net protein balance (de Boer et al., 2007; Ferrando et al., 1996; Kilroe et al., 2020). With prolonged unloading, there is a progressive activation of proteolytic pathways, including the ubiquitin–proteasome and autophagy–lysosome systems, leading to myofibrillar protein degradation (Dirks et al., 2018; Pang et al., 2023; Salanova et al., 2008). Consistent with this temporal sequence, reductions in muscle fibre cross‐sectional area are detectable after as little as 5 days of immobilization and progress with increasing duration of inactivity (Dirks et al., 2018; Hendrickse et al., 2022; Wall et al., 2014).

In parallel with altered protein turnover leading to muscle atrophy, strict physical inactivity induces pronounced metabolic remodelling of skeletal muscle. Reduced contractile activity lowers energetic demand while nutrient delivery is often preserved, resulting in intracellular nutrient overload, metabolic inflexibility and development of insulin insensitivity (Eggelbusch et al., 2024; Rudwill et al., 2018). Human bed rest studies demonstrate that insulin sensitivity declines within days of unloading (Dirks et al., 2016; Eggelbusch et al., 2024), associated with a rapid increase in intracellular glycogen and lipid storage (Eggelbusch et al., 2024). These acute impairments in insulin action may lead to a reduction in metabolic flexibility, the ability to efficiently switch between fuel sources depending on energy demand and nutrient availability, shifting substrate use toward greater glucose utilization and away from lipid oxidation (Eggelbusch et al., 2024; Smith et al., 2018).

At the mitochondrial level, physical inactivity rapidly leads to mitochondrial fragmentation (Eggelbusch et al., 2024), followed over time by reductions in mitochondrial density and oxidative capacity (Hendrickse et al., 2022). Rather than reflecting primary mitochondrial dysfunction, physical inactivity appears to be characterized by a loss of mitochondrial volume and network integrity, including mitochondrial fragmentation and reduced cristae density, accompanied by downregulation of oxidative phosphorylation proteins (Blottner et al., 2023; Eggelbusch et al., 2024). The mechanistic role of reactive oxygen species in these mitochondrial alterations remains unresolved. Bed rest studies suggest that although inactivity increases oxidative stress signalling, endogenous antioxidant systems largely prevent major oxidative damage to muscle proteins (Bosutti et al., 2026; Murgia et al., 2024). In contrast, other evidence suggests that oxidative stress may contribute to mitochondrial remodelling and changes in the skeletal muscle proteome during disuse (Murgia et al., 2024).

Satellite cells contribute to skeletal muscle maintenance and regeneration, and their function is suppressed during physical inactivity (Dirks et al., 2018; Relaix & Zammit, 2012). Human immobilization and bed rest studies generally report no reduction in satellite cell number, but an impaired satellite activation, as induction of myogenic regulatory factors such as myogenic differentiation 1 (MyoD) and myogenic factor 5 (Myf5) are blunted during immobilization (Dirks et al., 2018; Relaix & Zammit, 2012). Animal models of muscle unloading further show reduced satellite cell proliferation, fusion and myonuclear accretion with prolonged inactivity, suggesting compromised regenerative capacity (Liu et al., 2024; McCroskery et al., 2003). As such, satellite cells are unlikely to drive early disuse atrophy but can contribute to impaired muscle maintenance and delayed recovery upon myofibre damage after physical inactivity, bed rest or hospitalization.

Animal models of unloading, including hindlimb suspension and spaceflight analogues, closely recapitulate these human adaptations and confirm coordinated alterations in anabolic signalling, proteolysis and mitochondrial architecture (Hanson et al., 2023; McPherron et al., 1997; Murgia et al., 2024; Smith et al., 2020). Collectively, these findings indicate that inactivity‐induced muscle atrophy arises from an integrated failure of anabolic signalling, progressive activation of protein degradation pathways and metabolic alterations during deconditioning. The contributing molecular regulators that coordinate these maladaptive responses are currently not well understood. Among possible regulators, myostatin plays a central role in coordinating muscle protein synthesis, protein degradation and satellite cell activity (Hoogaars & Jaspers, 2018; McPherron et al., 1997). As such, myostatin presents itself as a key molecular player in skeletal muscle alterations during physical inactivity.

## 3 MYOSTATIN STRUCTURE AND FUNCTION

Myostatin is encoded by the *MSTN* gene on chromosome 2. It is a myokine synthesized within skeletal muscle fibres as pre‐pro‐peptide, which undergoes folding and proteolytic processing in the endoplasmic reticulum and Golgi apparatus. Subsequently, the latent pro‐myostatin complex is secreted into the extracellular matrix, preventing immediate autocrine inhibition of protein synthesis within the myocyte in which it was produced (Breitbart et al., 2011). Structurally, the myostatin precursor comprises a signal peptide, an N‐terminal pro‐peptide and a C‐terminal mature peptide. Proteolytic removal of the pro‐peptide is required to generate biologically active myostatin capable of receptor binding and downstream signalling (Wolfman et al., 2003).

**FIGURE 1:**
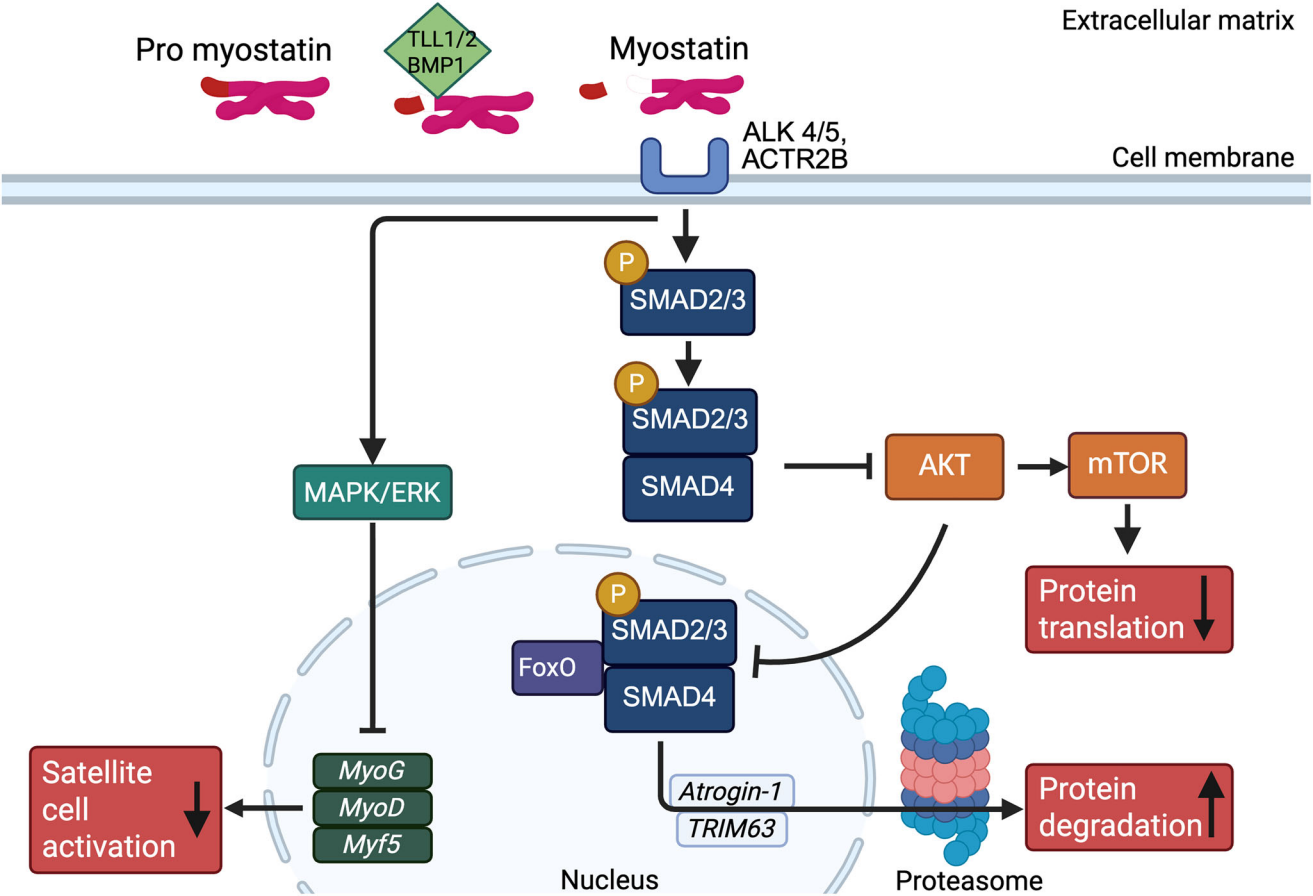
Canonical myostatin signalling in skeletal muscle. After proteolytic activation, mature myostatin binds to ActRIIB (ALK4/5) receptors, leading to phosphorylation of Smad2/3 and their nuclear translocation with Smad4. This signalling suppresses AKT1–mTORC1 activity, reducing protein synthesis, and enhances FoxO‐mediated transcription of atrogin‐1 (MAFbx) and muscle RING finger 1 (MuRF1/*TRIM63*), promoting ubiquitin–proteasome‐dependent proteolysis. In parallel, myostatin maintains satellite cells in a quiescent state by inhibiting MyoD and Myf5 expression.

The intracellular cascade of myostatin is summarized in Figure 1. Following proteolytic activation, mature myostatin binds to the activin type II receptor (ActRIIB) at the muscle cell surface, which recruits and phosphorylates the type I receptors activin receptor‐like kinase 4 and 5 (ALK4/5) (Wolfman et al., 2003). Activation of this receptor complex initiates canonical TGF‐β signalling via phosphorylation of Smad2 and Smad3, which subsequently associate with Smad4 and translocate to the nucleus (Hoogaars & Jaspers, 2018; Sartori et al., 2021; Wolfman et al., 2003). In skeletal muscle fibres, Smad2/3 signalling interferes with AKT1 phosphorylation, leading to suppression of mTOR activity and a consequent reduction in muscle protein translation (Hoogaars & Jaspers, 2018; Huang & Tindall, 2011; Sartori et al., 2009, 2021). Through this mechanism, myostatin reduces protein synthesis rates.

In addition, myostatin promotes skeletal muscle protein degradation (Baig et al., 2022; Hoogaars & Jaspers, 2018). Reduced AKT1 activity following myostatin‐induced Smad2/3 activation permits increased nuclear localization and activity of Forkhead box O (FoxO) transcription factors (Hoogaars & Jaspers, 2018; Huang & Tindall, 2011; Sartori et al., 2021). FoxO activation upregulates the gene expression of key components of the ubiquitin–proteasome system, including the muscle‐specific E3‐ubiquitin ligases F‐box only protein 32 (*FBXO32*) and tripartite motif containing 63 (*TRIM63*), encoding atrogin‐1 (MAFbx) and muscle RING finger 1 (MuRF1), respectively, thereby activating ubiquitination of myofibrillar proteins (Huang & Tindall, 2011; Pang et al., 2023). Through coordinated suppression of protein synthesis and activation of proteolysis, myostatin therefore drives a net negative muscle protein balance.

Beyond its effects on myofibre protein turnover, myostatin also regulates skeletal muscle regenerative capacity by maintaining satellite cells in a quiescent state (McCroskery et al., 2003). In satellite cells, Smad2/3 signalling suppresses the transcription of myogenic regulatory factors such as MyoD and Myf5, thereby inhibiting activation, proliferation and differentiation into myoblasts (McCroskery et al., 2003). Myostatin prevents the transition from the quiescent to the activated Myf5 and MyoD‐expressing state, limiting satellite cell activation (Liu et al., 2024). This suppression of satellite cell activation impairs fusion of satellite cells with myofibres, and ultimately, skeletal muscle growth, repair and regeneration (Dewasi et al., 2025; Liu et al., 2024; McCroskery et al., 2003; Schmidt et al., 2019).

## 4 INACTIVITY‐DRIVEN UPREGULATION OF MYOSTATIN

Alterations in the myostatin cascade are implicated in the molecular alterations upon physical inactivity. Reduced mechanical loading is known to increase *MSTN* gene transcription, leading to greater myostatin protein production and subsequent secretion of latent pro‐myostatin complexes into the extracellular matrix (Baig et al., 2022; Hoogaars & Jaspers, 2018; Lim et al., 2023; McPherron et al., 1997; Smith et al., 2020).

**FIGURE 2:**
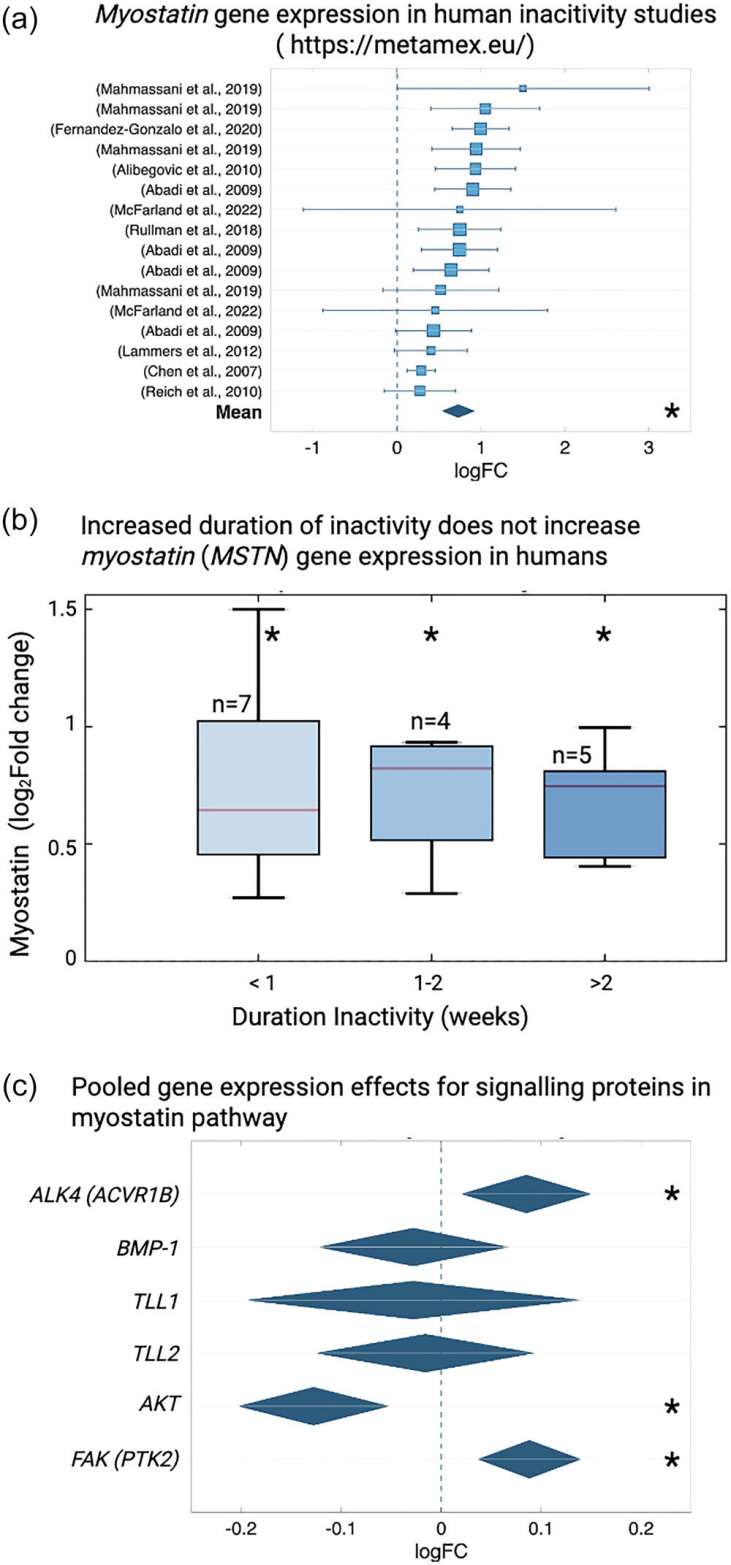
Myostatin (*MSTN*) expression in human inactivity studies (data analysis using MetaMEx database, metamex.eu). (a) Forest plot summarizing the effect of physical inactivity interventions on skeletal muscle myostatin (*MSTN*) mRNA expression across included studies. Each row represents an individual study (or study arm), with the square indicating the study‐specific effect size (log2 fold change; logFC) and the horizontal whiskers representing the corresponding 95% confidence interval. The vertical dashed line at 0 denotes no change versus the control/baseline condition; positive values indicate higher *MSTN* expression during inactivity. (b) Increased myostatin gene expression was not different between studies of different duration. (c) The pooled gene expression of proteins related to the myostatin signalling pathway showed higher expression of the ALK4 (ACVR1B) receptor and FAK (PTK2) in human inactivity studies, and reduced gene expression of *AKT1*. Other signalling molecules did not have altered gene expression upon inactivity in humans. The diamonds depict the pooled mean effect, with its width reflecting the 95% confidence interval. *P < 0.05 compared to zero. MetaMEx data retrieval on 23 February 2026.

Despite variability in the magnitude of the response, there is a clear consensus that physical inactivity is associated with upregulation of myostatin (Carlson et al., 1999; Dirks et al., 2018; Gustafsson et al., 2010; Irimia et al., 2017; Lim et al., 2023; Liu et al., 2024; Zachwieja et al., 1999). The temporal regulation of gene expression levels of myostatin and its associated molecular players during inactivity, however, remains incompletely understood. Nevertheless, changes in myostatin gene expression and protein levels can be detected as early as 3 days after the onset of inactivity (Gustafsson et al., 2010). Studies examining more prolonged immobilization report likewise elevated myostatin levels, although the magnitude of these changes varies considerably between studies, ranging from ∼12% (Zachwieja et al., 1999) to ∼60% (Lim et al., 2023) after 25 days of immobilization. Consistent with this, myostatin mRNA expression increased ∼1.6‐fold after 84 days of bed rest in healthy participants (*P* < 0.0001) (Irimia et al., 2017). To further evaluate inactivity‐associated transcriptional responses, we performed a time course analysis of myostatin gene expression (and related genes) during physical inactivity using publicly available transcriptomic datasets in the MetaMEx (https://metamex.eu/app/metamex) database (Pillon et al., 2020), which contains studies published up to August 2022 (Figure 2 and Table 1). With all studies included, we observed a ∼66% increase in myostatin (*MSTN*) gene expression (log_2_ fold change = 0.73; *P* = 5.10^−11^) across 16 different experiments (Figure 2a). The time course of *MSTN* gene expression in skeletal muscle showed a significant increase of ∼55% within 1 week of physical inactivity (*P* = 1.10^−3^), but there was no systematic increase in myostatin gene expression across inactivity studies with different durations (Figure 2b). The gene expression levels of ALK4 (ACVR1B) receptor increased across inactivity studies (Figure 2c). As ALK4 forms part of the myostatin receptor complex responsible for downstream Smad 2/3 activation, increased ALK4 expression may indicate an enhanced sensitivity of skeletal muscle to myostatin signalling during physical inactivity. This observation is consistent with the proposed shift toward suppression of anabolic signalling and activation of atrophy‐related pathways during unloading.

**TABLE 1:** Overview of studies included in the data analysis for Figure 2.

Study	GSE ID	Protocol	Muscle	Sex	Age	Body composition	Duration (days)
Mahmassani et al. (2019)	GSE113165	Bedrest	VL	M	Young	Lean	5
Mahmassani et al. (2019)	GSE113165	Bedrest	VL	F	Elderly	Overweight	5
Fernandez‐Gonzalo et al. (2020)	GSE148152	Bedrest	VL	M	Young	Lean	84
Mahmassani et al. (2019)	GSE113165	Bedrest	VL	M	Elderly	Overweight	5
Alibegovic et al. (2010)	GSE24215	Bedrest	VL	M	Young	Lean	10
Abadi et al. (2009)	GSE14901	Limb suspension	VL	F	Young	Lean	14
McFarland et al. (2022)	GSE186045	Bedrest	VL	M	Middle aged	Lean	35
Rullman et al. (2018)	GSE104999	Bedrest	VL	M	Young	Lean	21
Abadi et al. (2009)	GSE14901	Limb suspension	VL	M	Young	Lean	14
Abadi et al. (2009)	GSE14901	Limb suspension	VL	F	Young	Lean	2
Mahmassani et al. (2019)	GSE113165	Bedrest	VL	F	Young	Lean	5
McFarland et al. (2022)	GSE186045	Bedrest	VL	M	Young	Lean	35
Abadi et al. (2009)	GSE14901	Limb suspension	VL	M	Young	Lean	2
Lammers et al. (2012)	GSE33886	Limb suspension	VL	M	Young	Lean	21
Chen et al. (2007)	GSE8872	Limb suspension	GAS	U	Young	Lean	10
Reich et al. (2010)	GSE21496	Limb suspension	VL	M	Young	Lean	2

From the MetaMEx database (metamex.eu), accessed on 23 February 2026. GAS: gastrocnemius; VL, vastus lateralis.

The molecular cascade of events leading to the increased transcription of myostatin during inactivity remain poorly understood. Integrin‐focal adhesion kinase (FAK) signalling constitutes a primary mechanotransduction pathway in skeletal muscle (de Boer et al., 2007). It is anticipated that reduced FAK phosphorylation causes a lower AKT1 activity, and subsequent activation of FoxO transcription factors, shifting the intracellular signalling balance toward enhanced myostatin gene expression. While a direct transcriptional control of *MSTN* by FAK has not been demonstrated, reduced mechanotransduction signalling likely creates an environment for increased myostatin expression during physical inactivity. Our available data overview from MetaMEx suggests that pooled *AKT* gene expression was indeed reduced, but that FAK (*PTK2*) gene expression paradoxically increased after physical inactivity in humans (Figure 2c). Future studies should determine whether this increase in *FAK* gene expression marks a sensitization of the FAK signalling pathways with inactivity, and how its phosphorylation status relates to myostatin signalling.

Alternatively, it has been proposed that inactivity induces alterations in the extracellular matrix that indirectly promote myostatin activation (Dirks et al., 2018; Kilroe et al., 2020). These inactivity‐induced changes affect both extracellular matrix composition and the activity of myostatin‐cleaving enzymes (Breitbart et al., 2011; Dirks et al., 2018). Activation of latent myostatin requires sequential processing by a signal peptidase and extracellular proteases, including bone morphogenetic protein‐1 (BMP‐1) and Tolloid‐like proteases 1 and 2 (TLL1/2) (Wolfman et al., 2003). In the absence of mechanical loading, extracellular matrix turnover can become dysregulated (Kjaer, 2004), and results in collagen accumulation and increased extracellular matrix stiffness (Fix et al., 2021; Petrocelli et al., 2023). Remodelling of the extracellular matrix architecture could alter the spatial accessibility of activating proteases such as BMP‐1 and TLL1/2, potentially facilitating increased myostatin activation (Breitbart et al., 2011; Dirks et al., 2018; Pang et al., 2023; Wolfman et al., 2003). In our MetaMEx time course analysis, we did not observe significant differences in gene expression levels of these signalling molecules (Figure 2c).

It has further been suggested that increased extracellular matrix stiffness resulting from collagen accumulation may impair the clearance or diffusion of latent pro‐myostatin (Hosaka et al., 2012; Kjaer, 2004; Lee & McPherron, 2001). This could promote local retention and accumulation of latent myostatin within the extracellular matrix, thereby predisposing muscle tissue to a pronounced increase in myostatin activity upon subsequent activation or loading (Hosaka et al., 2012; Kjaer, 2004; Lee & McPherron, 2001). However, the precise effects of extracellular matrix remodelling on myostatin activation remain insufficiently supported by direct experimental evidence.

## 5 CONSEQUENCES OF INCREASED MYOSTATIN FOR SKELETAL MUSCLE DURING INACTIVITY

**FIGURE 3:**
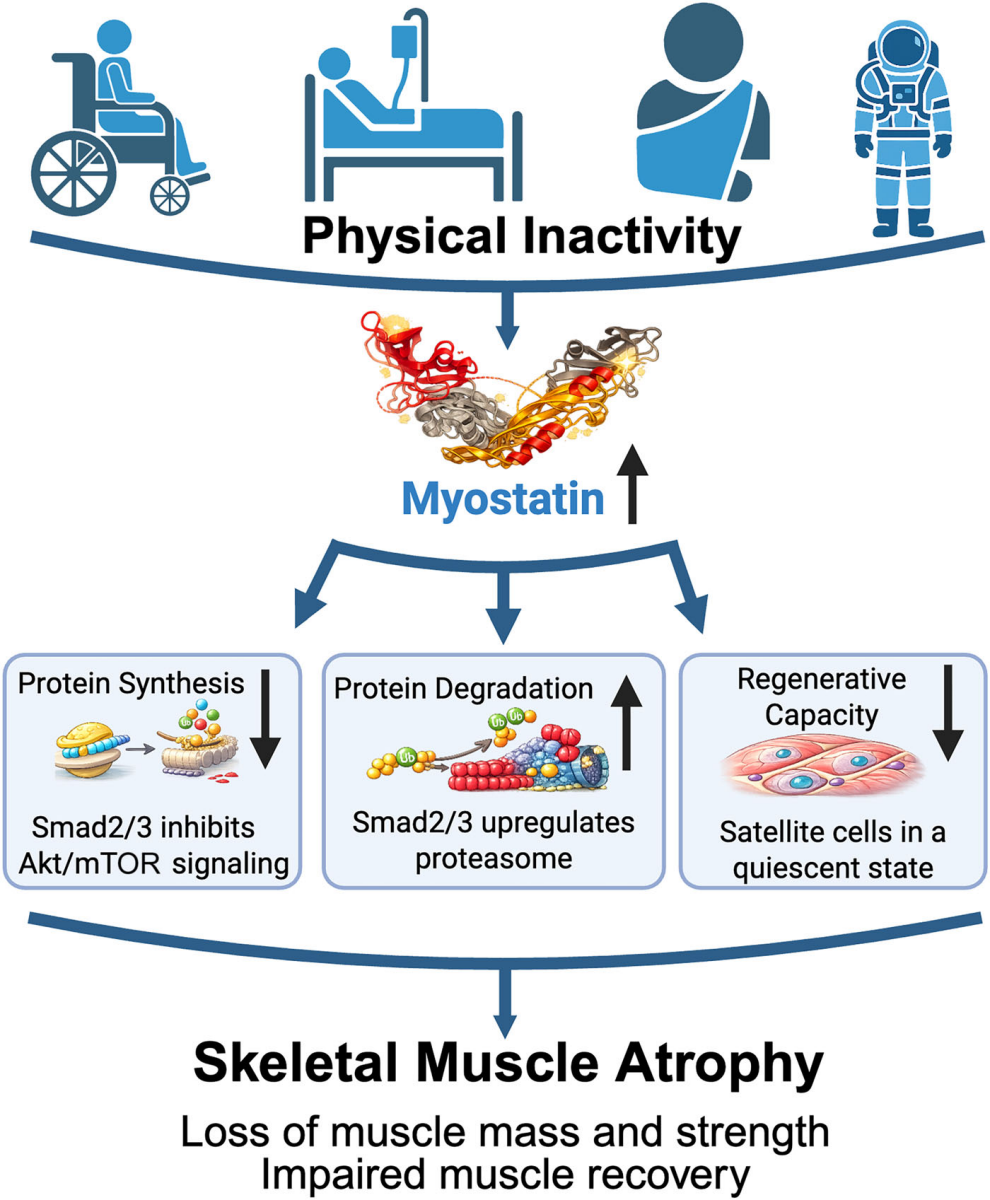
Physical inactivity‐induced myostatin upregulation and its contribution to skeletal muscle atrophy. Physical inactivity (e.g., immobilization, bed rest, limb casting or microgravity) increases skeletal muscle myostatin gene and protein expression, which ultimately activates canonical Smad2/3 signalling, suppressing Akt/mTOR‐mediated protein synthesis and promoting proteolysis. In parallel, increased myostatin signalling contributes to reduced regenerative capacity by maintaining satellite cells in a quiescent state and impairing myogenic activation. The combined effects of reduced protein synthesis, enhanced protein degradation and diminished regenerative potential result in net protein loss, leading to skeletal muscle atrophy characterized by decreased muscle mass and strength and impaired recovery following disuse.

Building on the evidence that inactivity increases myostatin levels, we propose here that this inactivity‐driven upregulation of myostatin serves as a central mediator of the progressive alterations observed in skeletal muscle, including muscle atrophy and reduced regenerative capacity. An overview of the proposed role of myostatin in inactivity‐induced skeletal muscle atrophy is presented in (Figure 3).

Evidence for the role of myostatin in determining muscle phenotype is illustrated by the case of a myostatin deficient infant, who exhibited a quadriceps muscle cross‐sectional area 7.2 standard deviations greater than age‐ and sex‐matched controls (Schuelke et al., 2004). Elevated levels of myostatin protein cause profound muscle and fat loss (Sartori et al., 2009; Trendelenburg et al., 2009; Wall et al., 2014; Zimmers et al., 2002), while inhibition of myostatin dramatically increases muscle size (Hanson et al., 2023; McPherron et al., 1997; Smith et al., 2020; Whittemore et al., 2003). While this observation highlights the potent influence of myostatin on muscle size, it does not provide direct insight into its mechanistic effects on skeletal muscle atrophy during inactivity. Increasing myostatin signalling induces muscle wasting, but myostatin is not the sole initiator of disuse atrophy. Inhibiting myostatin signalling by an anti‐myostatin peptibody (a phage display‐derived myostatin‐neutralizing peptide fused to a murine Fc domain) attenuates but does not fully prevent unloading‐induced atrophy in mice undergoing hindlimb suspension (Hanson et al., 2023). Particularly, the effects of myostatin on fibre size were muscle‐ and fibre type‐specific. In the predominantly fast‐twitch gastrocnemius, myostatin blockade increased fibre cross‐sectional area in the fast type IIx/b fibres toward control levels. In the predominantly slow‐twitch soleus, however, the same inhibition did not reverse inactivity‐induced fibre atrophy. On the molecular level, gene expression of *FBXO32* and *TRIM63*, encoding MAFbx (atrogin‐1)and MuRF‐1, respectively, was increased by hindlimb suspension, but myostatin inhibition did not significantly reduce their gene expression levels. Phosphorylated Akt tended to increase with myostatin inhibition, but this was not statistically significant. These findings highlight the therapeutic potential of myostatin targeting in disuse, but other molecular pathways beyond myostatin also play important roles in unloading‐induced atrophy.

In addition to regulating skeletal muscle size, myostatin also influences the regenerative capacity of muscle following injury or disuse (McCroskery et al., 2003). Myostatin maintains satellite cells in a quiescent state, inhibiting their activation and proliferation and preventing myofibre differentiation (McCroskery et al., 2003). This suppression impairs the regenerative response following periods of disuse, particularly when muscle damage occurs during reloading (Liu et al., 2024). Evidence from human muscle biopsy studies conducted after short‐term immobilization demonstrates both increased myostatin expression and elevated myogenin expression (Dirks, 2014). An increased myogenin expression is associated with higher MuRF1 (*TRIM63*) and atrogin‐1 (MAFbx, *FBXO32*) gene expression (Sartori et al., 2009) and altered satellite cell activation (Dirks, 2014). Myostatin signalling can trigger an atrophy programme in adult myofibres that is independent of MuRF1 and only partially dependent on atrogin‐1 (Sartori et al., 2009). Myostatin has also been shown to reduce MyoD expression and inhibit the differentiation of satellite cells, resulting in diminished muscle renewal (McCroskery et al., 2003; Schmidt et al., 2019). Similar findings have been observed in myostatin knockout mice, where delayed myogenin expression was reported (McCroskery et al., 2003). Together, these findings indicate that prolonged inactivity‐driven upregulation of myostatin not only accelerates skeletal muscle atrophy but also compromises the regenerative capacity of muscle tissue. This myostatin‐induced stabilization of atrophy signalling upon inactivity provides a novel mechanistic link between inactivity‐induced myostatin signalling and its detrimental effects on skeletal muscle structure and function.

## 6 CLINICAL APPLICATIONS

The evidence discussed thus far indicates that physical inactivity elevates myostatin levels and provides insight into the mechanisms through which myostatin contributes to skeletal muscle atrophy. Because myostatin is a potent negative regulator of skeletal muscle mass, pharmacological inhibition of myostatin signalling represents a potential promising strategy to mitigate skeletal muscle loss in situations where mechanical loading is limited or not feasible (Hanson et al., 2023). Therapeutic approaches include blockade of the ActRIIB–ALK4/5 receptor axis through decoy receptors or neutralizing antibodies (Morvan et al., 2017), as well as direct myostatin inhibition through binding proteins such as follistatin (Amthor et al., 2004; Morvan et al., 2017). Mechanistically, inhibition of myostatin signalling may alleviate anabolic resistance, by relieving Smad2/3‐mediated suppression of Akt–mTORC1 signalling (Figure 1), thereby restoring muscle protein synthesis and improving anabolic responsiveness of skeletal muscle to nutrients or mechanical stimuli (Trendelenburg et al., 2009).

Beyond the preservation of skeletal muscle mass during periods of inactivity, increasing skeletal muscle mass per se represents a metabolically meaningful therapeutic strategy. Global skeletal muscle hypertrophy has been shown to exert anti‐obesity and anti‐diabetic effects, whereas muscle atrophy promotes fat accumulation and worsens glucose homeostasis (Guo et al., 2009; McPherron et al., 1997; Rovira Gonzalez et al., 2019). These reciprocal relationships highlight that skeletal muscle mass is not merely a functional tissue but a key regulator of systemic metabolic health. Recent work further suggests that myostatin functions as a molecular link between skeletal muscle dysfunction and metabolic disease, as elevated myostatin signalling has been associated with insulin resistance, chronic inflammation and extracellular matrix remodelling in metabolically compromised muscle (Cesanelli et al., 2026). In this context, the concept of ‘turning fat into muscle’ has been proposed as a body composition‐focused therapeutic paradigm, whereby increasing muscle mass or preventing atrophy may improve metabolic homeostasis independently of weight loss alone (Wackerhage et al., 2024). Translational evidence in humans further supports this concept. Pharmacological blockade of activin type II receptors with bimagrumab increased lean mass while significantly reducing fat mass and glycated haemoglobin levels in individuals with obesity and type 2 diabetes mellitus (Heymsfield et al., 2021). More recently, the phase 2 BELIEVE trial demonstrated that combining bimagrumab with the glucagon‐like peptide‐1 (GLP‐1) receptor agonist semaglutide produced greater body weight reductions than either treatment alone, supporting the potential of fat‐to‐muscle repartitioning strategies as a complement to established obesity pharmacotherapies (Heymsfield et al., 2026). Therefore, such fat‐to‐muscle repartitioning strategies, whether achieved through resistance training, nutritional interventions or targeted pharmacological approaches such as myostatin inhibition, offer clinically meaningful benefits for individuals with obesity or metabolic disease.

These myostatin‐inhibiting strategies hold potential for counteracting skeletal muscle atrophy during periods of inactivity associated with hospitalization (Kangalgil et al., 2024; Zachwieja et al., 1999), spaceflight (Smith et al., 2020) or limb immobilization (Lim et al., 2023), where a similar muscle‐to‐fat transition occurs (Eggelbusch et al., 2024). Muscle atrophy in these contexts has important clinical implications, as patients with low skeletal muscle mass during critical illness or hospitalization are at significantly increased risk of mortality (Yang et al., 2023). Moreover, muscle wasting during hospitalization is associated with impaired physical recovery (Mayer et al., 2020), increased susceptibility to infections, greater insulin resistance and a higher incidence of adverse clinical outcomes (Dirks et al., 2016).

However, the precise regulation and context‐specific signalling mechanisms of myostatin in humans are incompletely understood, and its causal contribution to disuse‐induced skeletal muscle atrophy is yet to be fully delineated. Addressing these gaps is critical, since improved mechanistic insights will aid in guiding the development of potential therapeutic strategies and clinical applications targeting myostatin signalling.

## 7 LIMITATIONS AND FUTURE PERSPECTIVES

Although the literature consistently demonstrates that physical inactivity is associated with increased myostatin gene and protein expression, several important limitations temper strong mechanistic conclusions and provide directions for future research. Most importantly, a complete causal molecular mechanism in humans is still lacking. Current evidence supports the interpretation that myostatin behaves as part of a ligand‐level adjustment within a broader Smad2/3‐controlled transcriptional network rather than as a uniquely regulated gene driven by a single upstream factor (Laskin et al., 2024). Functional redundancy within the TGF‐β/activin ligand–receptor network reinforces this view, as Smad2/3 signalling might be viewed as the central signalling hub, with myostatin being one of several upstream inputs (Lee et al., 2005; Sartori et al., 2009, 2021; Winbanks et al., 2012).

The temporal profile of myostatin regulation during inactivity remains poorly defined. Although most studies report increased myostatin gene expression or protein levels during bed rest or immobilization, substantial variability exists regarding magnitude and timing. It remains unclear when myostatin and its upstream regulators and downstream effector proteins peak, and whether myostatin plateaus during prolonged inactivity, or declines to a new steady state. Particularly the role of FAK signalling during inactivity represents an understudied topic. Carefully staged serial biopsy studies (e.g., 1–3, 7, 30 and/or ≥60 days), would provide essential insight into early mechanotransduction‐sensitive events versus later stabilization phases. Novel ‐omics techniques, such as phospho‐proteomics or other ‐omics methods focused on post‐translational modifications, will need to be used to fully understand the role of myostatin in skeletal muscle structure and function.

One limitation in the currently available body of research is that some studies assess circulating, rather than skeletal muscle, myostatin levels (Lim et al., 2023; Zachwieja et al., 1999). While plasma measurements provide useful information on systemic myostatin levels, they offer limited insight into local muscle‐specific effects, extracellular matrix remodelling and fibre type‐specific responses. These factors are considered critical for understanding the full impact of inactivity‐induced myostatin upregulation on skeletal muscle (Ahmad et al., 2023; Baig et al., 2022).

Nutrient overload during physical inactivity offers another promising avenue for upstream myostatin regulation. Bed rest studies demonstrate rapid insulin insensitivity and lipid accumulation (Eggelbusch et al., 2024), which likely affect Akt phosphorylation and activity. Given the crosstalk between Akt signalling, FoxO activation and Smad pathways, combining measurements of insulin sensitivity and anabolic resistance, together with molecular profiling of Akt, FoxO and Smad signalling, would clarify whether early metabolic inflexibility induces myostatin signalling or whether myostatin contributes to the development of anabolic resistance and insulin insensitivity. Indeed, experimental evidence suggests that complete myostatin deficiency may impair the normal mechanical adaptation of skeletal muscle to chronic overload, indicating that basal myostatin signalling may be required for optimal contractile and structural adaptation to increased loading (Cesanelli et al., 2025).

Recent work has highlighted that myostatin exerts systemic endocrine effects beyond skeletal muscle. Recent data implicate myostatin in the regulation of follicle‐stimulating hormone synthesis and the hypothalamic–pituitary–gonadal axis, raising the possibility that systemic inhibition could negatively influence reproductive function (Ongaro et al., 2025). Therefore, future translational studies involving myostatin‐targeted therapies, such as bimagrumab, should incorporate comprehensive endocrine phenotyping, including reproductive hormones, to ensure safety, particularly during prolonged treatment.

## 8 CONCLUSION

Inactivity‐induced elevations in myostatin contribute substantially to the loss of skeletal muscle mass. This effect is mediated through three primary mechanisms: suppression of protein synthesis, upregulation of protein degradation pathways and inhibition of satellite cell activation. These processes are tightly orchestrated by myostatin signalling. Although not all mechanisms underlying inactivity‐induced myostatin upregulation are fully understood, further elucidation of myostatin's role holds significant potential for improving human health, physical performance and recovery across a range of contexts, from clinical rehabilitation to long‐duration spaceflight. Balancing therapeutic efficacy with systemic safety will be essential for the successful clinical application of myostatin inhibition strategies.

## AUTHOR CONTRIBUTIONS

D.N. wrote the first version as part of the course ‘Exercise and Clinical Immunology’ for the Research MSc Human Movement Sciences at the Vrije University Amsterdam. All authors contributed to revising it critically for important intellectual content. All authors approved the final version of the manuscript, agreed to be accountable for all aspects of the work in ensuring that questions related to the accuracy or integrity of any part of the work are appropriately investigated and resolved, and all persons designated as authors qualify for authorship, and all those who qualify for authorship are listed.

## CONFLICT OF INTEREST

None declared.

## GENERATIVE AI STATEMENT

ChatGPT (GPT‐5.5 Thinking; OpenAI) was used for language polishing and restructuring text as none of the authors is a native English speaker. The tool was used in a manner that does not conflict with ethical policies, and the authors take full responsibility for the content.
